# Parent organizations’ experiences of the pandemic response in maternity care in thirteen European countries

**DOI:** 10.18332/ejm/156902

**Published:** 2022-12-22

**Authors:** Daniela Drandić, Katharina Hartmann, Catarina Barata, Rosa Torguet

**Affiliations:** 1 Roda - Parents in Action, Zagreb, Croatia; 2 Human Rights in Childbirth, United States; 3 Mother Hood e.V., Bonn, Germany; 4 Institute of Social Sciences, University of Lisbon, Lisbon, Portugal; 5 Portuguese Association for the Rights of Women in Pregnancy and Birth, Lisbon, Portugal; 6 El Parto Es Nuestro, Alicante, Spain

**Keywords:** maternity care, COVID-19, crisis response, epidemiological measures, mental health, postCOVID-19 policy

## Abstract

We surveyed changes to maternity care services in the first 17 months of the COVID-19 pandemic in 13 different European countries, from the perspective of national maternity service (parent) organizations advocating for a human rights approach to maternity services. A qualitative study was conducted in November 2020. An open-question survey was sent to national maternity service (parent) organizations and members of COST Action 18211 in Europe, asking about COVID-19 measures in maternity services (antenatally, intrapartum, postnatally, and overall satisfaction). From the open answers, 16 core issues were extracted. Between February and August 2021, semi-structured interviews with the national representatives of 14 parent member organizations in Europe were conducted, collecting details on overall national situations and changes due to COVID-19 measures. The reported experiences of parent organizations from 13 European countries show wide variations in epidemiological containment measures during the first 17 months of the COVID-19 pandemic. Practices differed between facilities, resulting in emotional disquiet and confusion for parent-patients. Most countries maintained antenatal and postnatal care but restricted psychosocial support (antenatal and birth companions, visitors). Organizations from nine countries reported that women had to wear masks during labor, and all but two countries saw separations of mothers and babies. Most parent organizations described a need for more reliable information for new parents. During the pandemic, non-evidence-based practices were (re-) established in many settings, depriving women and families of many factors which evidence has shown to be essential for a positive birthing experience. Based on the findings, we consider the challenges in maternity services and propose a strategy for future crises.

## INTRODUCTION

Since the beginning of the pandemic, human rights, feminist, patient, and maternity advocacy groups have voiced their concern over fundamental rights violations in maternity care^1^. Early in the pandemic, COVID-19 was identified as a risk factor for obstetric violence^2^, often accompanied by rollbacks in quality of care^3-5.^ In their commentary, our colleagues from COST Action 18211point^5^ points to mounting evidence of adverse consequences that COVID-19 containment measures enforced in facility maternity care worldwide. They call for the priority implementation of evidence-based, human-rights-informed care, including during times of crisis such as the current pandemic. The commentary authors go on to highlight how the reaction to health services and the way they were reorganized reveals ‘something about the underlying ethos of maternity care provision around the world, raising serious questions about how it should be reframed when services are rebuilt once the pandemic is finally over’^5^.

Patient advocacy organizations, in this case, national parents’ groups monitoring and advocating for improvements in maternity services, are relevant stakeholders in healthcare^6^ because they work at the intersection of parents’ lived experiences, policies and rights, using their unique insight to catalogue and report patients’ lived experiences in general^7-9^, but also in maternity services specifically^1,10,11.^

As in any crisis, caution is advised when reactions include sudden practice changes to maternity services that are not grounded in the best available evidence. These can bring unforeseen consequences for women, pregnant and birthing people, and families. Respectful maternity care cannot be seen as secondary to pandemic containment measures in maternity facilities; it is imperative that all containment measures are proportionate to the threat (in this case, the spread of COVID-19) and that undue burdens are not put on pregnant, birthing, and postnatal families. Practice guidelines for maternity care during COVID-19 in Europe during the first three waves of the pandemic, in countries where they existed, revealed erratic, contradictory and inconsistent approaches to scientific evidence^12^. Moreover, there is emerging evidence that containment measures implemented in maternity services as a response to the COVID-19 pandemic negatively impacted maternal and perinatal outcomes ^13,14^, including maternal mental health^15-17^ but also healthcare professionals’ well-being^18^.

Although some authors have reported on parents’ experiences in neonatal care ^11,19^, to our knowledge, no other published research has used a human-rights-based approach to survey national pandemic-related changes to care for pregnant and birthing women from the perspective of parents’ organizations. Organizations representing patients overall, or specific patient groups (e.g. cancer patients) have been conducting research and publishing reports using similar methodology throughout the pandemic^7-10 .^ The perspective of patient organizations, who collect and monitor information from patients receiving care directly but also through monitoring activities (e.g. social media, policy monitoring) is unique and, in a crisis, is a valuable source of real-time information.

This article aims to provide an overview of different changes made to maternity services implemented in some or all maternity facilities over the first 17 months of the pandemic, roughly corresponding to the first three waves of the pandemic in Europe, as reported by maternity service (parent) organizations who are monitoring the situation in their countries. The article reports on 16 core services and containment measures implemented in maternity services which directly impacted women, birthing people and families. Based on the findings, we propose a strategy for reimagining post-pandemic maternity services across Europe, including these 16 issues and consider the implementation challenges.

## STRATEGY FOR POST-PANDEMIC MATERNITY SERVICES

We used a descriptive qualitative and participative approach to gather input from parents’ organizations across Europe by preparing the protocol, research questionnaire and semistructured interviews in a participative manner. According to Sandelowski^20^, a ‘Qualitative description is especially amenable to obtaining straight and largely un-adorned [...] answers to questions of special relevance to practitioners and policymakers’.

In November 2020, a poll of COST Action 18211 network members was conducted online with six questions that collected information on the most significant changes in maternity services during the pandemic. Seven COST member organizations completed the survey, identifying some of the major COVID-19 containment measures implemented in their countries, which women and families had reported to them as problematic. The authors compared and analyzed the survey responses by clustering the data according to the phases of provision of obstetric care (antenatal, intrapartum and postnatal care), which identified 16 core issues from the survey for national maternity (parent/patient) organizations, as detailed in Table 1.

**Table 1:** Reports on maternity care during the height of the first, second and third waves of COVID-19 in 13 European countries

	*Portugal*	*Spain*	*Ireland*	*UK*	*Netherlands*	*Germany*	*Italy*	*Poland*	*Czechia*	*Slovakia*	*Hungary*	*Croatia*	*Cyprus*
**Antenatal care**													
In-person appointments as normal	(✓)	(✓)	✓ (+) 15 min maximum	✓	✓	✓	✓	✓	✓	x* / ✓	(✓)	✓	✓
Companion at antenatal appointment	x	x	x	x	(✓)	x	x	x	(✓)	x	x	-	x
Access to routine tests and scans	(✓)*	(✓)	✓ (+) 15 min maximum	✓	✓	✓	✓	✓	✓	(✓)* / ✓	✓	✓	✓
Antenatal mental health services available	-	- / x	✓	Reduced	(✓)	-	-	-	-	-	-	-	-
**Intrapartum care**													
Companion at vaginal birth	x* / ✓**	(✓) / ✓**	✓**	✓**	✓	✓* / ✓**	x* / (✓) / (✓)**	x	✓*	x* / (✓)	✓** / x	x	✓** / ✓*
Companion at CS birth	x / -	x	✓ (+)	(✓) / x	✓	✓	x / -	x / -	(✓)	-	-	-	✓ (only in private hospitals)
Doula support allowed	x / - (most hospitals only allowed one companion)	(✓)	x	x	(✓)	x	x / -	x	x / - (most hospitals only allowed one companion)	x	x	x	x / -
Women required to wear masks during labor and birth	✓	✓	x	x	x	(✓)	✓	Only COVID-19+ women	x	✓ (FTP2)	✓	✓	✓
Visitor bans	✓	✓	✓*	✓	(✓)	✓	✓	x	x	x	✓	✓	✓
Reports of women suspected or confirmed Covid-19+ being induced	(✓)	x	x	x	x	x	No information	x	x	No information	x	(✓)	x
Reports of women suspected or confirmed Covid-19+ having CS	(✓)	x	x	x	x	(✓)	No information	✓	x	Some reports, no information on scale	(✓)	(✓)	✓
Home birth services	Available, private	Available, private	Available, increased due to demand	Available, reduced	Available	Available	Available, private	Available, private	-	-	Available, private	-	-
**Postpartum care**													
Separation of COVID-19+ or suspect mother and baby	(✓)	✓	x	✓	x	(✓)	✓* / ✓	✓	✓	✓	(✓)	(✓)	✓ (until November 2020)
Visiting bans or time restrictions for premature or sick babies and parents	(✓)	✓ (+)	✓ (+)	✓ (+)	x	✓	✓ / -	1st wave: ✓* Later waves: ✓ (+)	✓	x* / (✓)	✓	✓	✓* / (✓)
Postpartum mental health services available	-	✓	✓	Reduced	✓	If available, difficult to access	-	-	-	-	-	-	-
In-person postpartum care (6-week appointment) available	✓	(✓)	✓ (+) 15 min maximum or via video-call	x or via phone	✓	(✓)	✓ / -	✓	✓	(✓)* / ✓	✓	✓	✓

✓ Practice at most facilities. (✓) Practice at a minority of facilities. x Not a practice at most facilities. - Not a practice/service in pre-COVID-19 times. *Ban in place during the first wave, but not later. **Companion only allowed at the end of birth, pushing phase. (+) Restrictions and time limits were introduced. / Delimiter between the waves.

Based on these results, we prepared a questionnaire with the 16 core issues and conducted semi-structured interviews with representatives of national maternity service (parent) organizations, recruited through COST Action 18211 and the European Network of Childbirth Associations (ENCA). Five of the organizations from the COST Action had also participated in identifying the 16 core issues, while the others did not. The interviewees were invited to comment on the identified issues and to detail the situation in their countries, based on reports they had received from parents on the ground and information they had gathered nationally. The focus was on generalized tendencies observed in the individual countries, leaving space for single (major) facilities or regions differing from this overall trend. The information gathered through the interviews is given in Table 1. Upon completion of an interview, the information was shared with the interviewees, who were invited to review and revise the recorded responses if needed.

Data collection, processing and storage, conformed to the General Data Protection Regulation (GDPR) and the Declaration of Helsinki. Consent was given at the beginning of each interview, with the possibility of exiting the interview at any time. No financial or other incentives were offered to the interviewees.

Table 1 summarizes the data collected during the semi-structured interviews for each country. A discussion on general trends across the countries continues below.

### Antenatal care

Interviewees reported that in-person antenatal care appointments were reduced overall across the countries studied, with the change being quite drastic in some countries. In Ireland, antenatal and postnatal visits were capped at 15 minutes regardless of the reason for the appointment, and in Slovakia, in-person appointments were banned during the first pandemic wave. Routine tests and scans remained available but were affected by shorter appointment times (Ireland) and were difficult to access during the peaks of the first three COVID-19 waves (Portugal, Spain, Slovakia).

Companions at antenatal appointments were banned in most countries, except the Netherlands and the Czech Republic, where interviewees stated that some facilities allowed companions. One country (Croatia) did not have the practice of companions at antenatal appointments prior to the pandemic. Overall, interviewees commented that the quality of care was compromised as a result, especially in countries where the quality of care was already low or varied before the pandemic. Antenatal mental health services, where they existed prior to the pandemic, were reduced. This is particularly problematic considering that the pandemic triggered the compromise of the mental health of all people, especially pregnant, birthing, and postnatal families^21,22^.

Parents’ organizations reported observing higher stress levels among pregnant women in their countries because of these difficult circumstances. One organization quoted a pregnant woman’s experience in Germany:

*‘My gyn offered me this test for malformations and my partner was not there but I just did not know what to do and how could I decide this without asking his opinion? It is his baby, too’*.

### Intrapartum care

*Companionship*

Parents’ organizations reported differences in the possibility of having a birth companion. This measure was subject to the broadest variety over time, type of facility and country. All interviewees reported a reduced possibility of having a birth companion or the institution of outright bans. Another experience from Germany showed the rapidly changing situation:

*‘The ward that allowed my partner with me was closed the day before I gave birth because they focused staff on COVID patients. I had to go to a hospital with a very different policy where my partner stayed in the parking lot the whole time’*.

In those situations where a birth companion was allowed, interviewees reported severe restrictions, including limiting when the companion was allowed to be present or the requirement that the birth companion have a negative COVID-19 test, which before the advent of rapid tests usually meant a minimum 24-hour wait. Only one country (Netherlands) stood out for being consistent in maintaining the same level of a right to a birth companion at a vaginal birth throughout the pandemic.

Women who give birth by cesarean section are not always afforded the same rights to companionship as those who give birth vaginally. Prior to the pandemic, reports by interviewees showed that the situation regarding companionship at a cesarean varied widely across the countries. In three countries, interviewees reported having a companion at a cesarean section was not the norm before the pandemic (Slovakia, Hungary, Croatia). In four countries where companionship at a cesarean was mostly the norm prior to the pandemic, bans were introduced as part of COVID-19 containment measures (Portugal, Spain, Italy and Poland), with differences between public and private facilities in Portugal, even though companionship at cesarean section is guaranteed by law^23^.

Doulas have appeared as second-birth companions over the past twenty years across Western Europe. Despite the overwhelming evidence that the presence of a doula can improve birth outcomes for mothers and newborns, especially for racialized and poor women, migrants and refugees^24-26^, interviewees reported that second companions, and therefore doulas were banned as part of containment measures in the majority of the countries included in the survey, with only two countries offering the possibility of doula companionship in some facilities (Spain, Netherlands).

Visitor bans were reported by most parents’ organizations in the study, whereby women were not able to see their partners or other family members after birth and for the duration of their stay in-hospital, at least during the peaks of COVID-19 waves. The exception to this were three countries (Poland, Czechia, Slovakia). Later research linked these types of bans to increased postnatal traumatic stress response^21,27^. It is important to note that this study did not collect data on the length of the average hospital stay after vaginal or cesarean birth, which may vary across the countries and alter the long-term effects of visitor bans.

*Mode of birth*

While most parents’ organizations surveyed did not report that women who were suspected of having or were COVID-19 positive (COVID-19+) had their labors induced without obstetric indication, in two countries (Portugal and Croatia) there were reports of this happening in some facilities. In two countries, interviewees reported that most facilities required that women with suspected or confirmed COVID-19 give birth by cesarean section without obstetric indication (Poland, Cyprus). In Poland, women who were COVID-19+ were required to birth in special facilities, where cesarean section was mandatory for COVID-19+ mothers. In five other countries, interviewees reported mandatory cesarean for COVID-19+ women for some facilities (Portugal, Germany, Italy, Hungary, Croatia), during certain pandemic waves. This echoes results published for the United States, which showed increased medicalization of childbirth for women suspected or confirmed of having COVID-19^28^.

*Availability of home birth services*

During the pandemic, more women sought home birth services^29^. In this study, organizations in six surveyed countries stated that only private-sector home birth services continued to be available as normal during the pandemic. In Portugal, where home births are only offered in the private sector, their number nearly doubled in 2020 compared to 2019^29^, which can partially be explained by people wanting to avoid the restrictions implemented in facilities, and perceived safety in avoiding COVID-19 infection. As the demand increased substantially, some women were not able to find midwives who could attend a home birth.

Two organizations stated that home birth services remained available as normal in both public and private sectors (Netherlands, Germany). Interestingly, the Irish parent-organization reported that the health service increased the availability of home birth services as a result of increased demand. In four of the countries, organizations reported that home birth services were not available before the pandemic, and were therefore not available during the pandemic either (Czechia, Slovakia, Croatia and Cyprus). Despite not being officially available, the number of home births in Croatia increased by 30% between 2019 and 2020, although the number remained low^30^.

*Separation from newborn and visiting bans*

Early in the pandemic, the WHO issued recommendations clearly stating that if the mother is COVID-19+, the mother and newborn should be kept together, provided the mother feeling well enough to care for the newborn^31^, a recommendation mirrored by the guidelines by the Royal Colleges of Midwifery and Obstetrician-Gynecologists^32^. Despite this, one of the major problems with COVID-19 response policies was the separation of mothers who were suspected or confirmed COVID-19+ and their newborns, especially in the first three to nine months of the pandemic; this was reported by most interviewees. Notable exceptions to these were reports from organizations from Ireland and the Netherlands, which stated that mother–newborn dyads were kept together as normal, regardless of the mother’s COVID status.

In some countries, the health authorities were slow to issue evidence-based recommendations. In Portugal, the first recommendation by the College of Obstetrics and Gynecology in March 2020 stated that women who are COVID-19+ must be separated from their infants and prevented from breastfeeding^33^. As more evidence and international guidelines were published resulting in increased pressure from advocacy groups, the General Directorate of Health updated the guidelines, albeit one full year later (March 2021). Despite the new guidance, the previous policies continued across facilities^34^. In Croatia, women who gave birth before the results of their routine PCR test were available, were separated from their newborns as a precautionary measure until a negative result was returned. This practice was prevalent in some facilities throughout the pandemic waves but also between waves, and was especially a problem in smaller facilities where PCR tests took longer to analyze.

Visiting restrictions for premature or sick newborns were also reported by most national organizations, with some interviewees reporting the implemented total visitation bans in most facilities (Czechia, Hungary, Croatia, and Cyprus). These measures were implemented despite a lack of evidence that they contribute to containing the spread of COVID-19 and despite being in contradiction with readily available professional and WHO guidelines that were being updated in real-time^32,35^.

Visitor bans were also reported for hospitalized mothers and newborns by all interviewees except those from three countries (Poland, Czechia, Slovakia), where parents’ organizations reported that visitation of mothers was the same as in pre-pandemic times. In the other countries, organizations reported that women hospitalized during pregnancy did not have visitation from the time of their hospitalization to post-partum, regardless of the duration of that stay (from two days to several weeks, in the case of complications). A mother from Germany stated:

*‘I stayed at the hospital because of late pregnancy complications but after my cesarean birth I left as soon as they let me - I missed my older daughter so much! I hadn’t seen her in 2 weeks’*.

Evidence shows that these visiting restrictions can have a negative effect on postnatal mental health^14,21,36^.

### Postnatal maternal mental and physical health

Despite increasing awareness about the importance of perinatal mental health during the pandemic, interviewees reported that mental health support services were only rarely available, either because they did not exist pre-pandemic, because they were restricted due to containment measures or because demand was so high that services were not accessible.

Interviewees reported that in-person postnatal care, usually organized around six-weeks after birth continued to be mostly available as normal, much as antenatal care was. However, they also reported that pandemic measures resulted in increased use of telehealth consultations, which depend on women and families having access to reliable, affordable internet and devices capable of video calls, which is not always possible. Later evidence about the efficacy of telehealth maternity care during the pandemic showed that this type of care was not always optimal^5,37^.

The results we found were similar to those described by others^19,38,39^, who defined similar themes: less family involvement, reduced emotional and physical support for women, compromised standards of care, increased exposure to medically unjustified cesarean section, and staff overwhelmed by rapidly changing guidelines and enhanced infection prevention measures^40^, as well as a decrease in respectful care due to fears surrounding COVID-19 transmission^2,38,41^. At the same time, interviewees reported that facilities that were known for providing more mother-friendly and baby-friendly care made great efforts to maintain that level of quality, sometimes at considerable staff effort, whilst otherwise less friendly facilities more rapidly downgraded their services.

### Maternity care guidelines from international and national organizations

Throughout the pandemic, international and national organizations have made efforts to create and regularly update guidelines for COVID and maternity care starting. Even the European Parliament was concerned about rollbacks in maternity care in member states^42^. Despite these efforts, our data have shown that facility policies did not change much between the first and third waves of the pandemic, even after more evidence was available.

A specific example is the benefits of labor and birth companions, which have been widely studied and recognized^22,24^. Guidance available very early in the pandemic and updated in real-time as new evidence became available^31,32^ clearly stated that companionship at vaginal birth was important and could safely continue with the implementation of basic pandemic practices. Despite this, the political will to maintain proportionally appropriate limitations to companionship among policymakers and professional organizations varied widely throughout the various countries and pandemic waves. An example of good midwifery practice was the UK during the second wave, where professional organizations reiterated that attendance of partners during labor and birth must be a priority, with reliable testing and appropriate PPE available to both, ensuring the safety of all those using maternity services, and must be proportionate to policies being used in other departments of the facility^32,43^.

A second good practice came from France, which did not have a parents’ organization representative included in this study but is a major European country. There, the Ministry of Health issued guidance for hospitals in the first month of the pandemic outlining how to include companions in labor and childbirth, while lowering the risk of infection^44^. The German Society for Gynecology and Obstetrics issued a similar statement^45^ on the inclusion of fathers, also in the first month of the pandemic.

The real-time guidelines by the Royal Colleges of Obstetrics and Gynecology and the Royal College Midwives and World Health Organization, referred to earlier in this article, stated that elective induction or cesarean should be avoided for women who have symptoms of or are positive for COVID-19, without obstetric indication^31,32^. Other European professional societies issued similar statements, including the German Obstetric Society^46^ and the Italian Obstetric Society^47^. Conversely, the Portuguese College of Obstetrics and Gynecology recommended a shorter threshold for the decision in the use of epidural analgesia and instrumental birth, meaning that women were more likely to have either of these interventions if they were suspected of or confirmed to be COVID-19+^33^.

### Maternity evidence-based human-rights care

The differing responses to the COVID-19 crisis have shown that national and local decision-makers’ opinions often impact maternity facility policies more than scientific evidence and international guidelines. As a result, there must be a significant change to the prevailing paradigm and overmedicalization of maternity care in Europe, especially during crises, to one where a proportional, evidence-based response prevails. A healthier perinatal period sets babies and families on a long-term beneficial health trajectory^48^. So, while ‘classic’ impediments to system changes such as financial and human resources must not be underestimated, it seems that the biggest challenge to good quality services lies in an ethical decision: ‘Is the well-being, physical and mental health of mothers, babies, and families important to our societies?’; and ‘Is an evidence-based, human-rights informed, salutogenic approach to maternity care politically desired?’. If so, policymakers need to take appropriate steps to enforce such an approach, as the current political and medical establishments alone seem unable or unwilling to birth the necessary change.

A woman-centered, positive birth experience (with no unnecessary interventions, in a space that feels physically and emotionally safe to the mother) is a valuable, long-term investment for any society. These birth experiences protect the physical and mental health of mothers^49^, and result in higher breastfeeding rates^50^, which should be an integral part of public health planning.

### Limitations

All the countries included in the survey have a mix of public and private facilities and insurance, which may have had different policies during the peaks of the pandemic waves. These have been noted where appropriate, but the mix of public and private facilities varies widely across the countries. Additionally, the results reflect general national trends, as they were reported to and collected by parent (patient) maternity rights organizations with a national presence in their countries. Although most of the organizations are large, experienced organizations with national networks, there is a potential for reporting bias. The multi-national nature of the data collection and importance of collecting these experiences, which may never be reported in official data, make them a valuable source of information. Future quantitative research will shed more light on the nuance in changes in maternity services during the pandemic. Furthermore, the research questions did not take into consideration access to medical abortion or medically assisted fertility services, and therefore did not map these aspects of reproductive healthcare. Finally, the research only includes information for the first 17 months of the pandemic, roughly corresponding to the first three COVID-19 waves in Europe, with data collection happening from May to July 2021, and for this reason does not include information on vaccines, rollout to pregnant individuals or data on the fourth wave, which began in Europe in September–October 2021.

## CONCLUSION

Crises such as pandemics are a litmus test for health services and societies, requiring a balance between containment measures and quality care. The responses to the COVID-19 pandemic mapped in this article have shown that in some countries, pregnant, birthing and postnatal women and their families were expected to bear a disproportionate burden of the pandemic response. From a parents’ perspective, the solution lies in a paradigm change, towards a respectful, woman-centered and family-centered approach^51^. The prevailing biomedical model of maternity services focused on pathology, must give way to a neuro-psycho-social model of care where maternity care services are shaped according to woman’s needs, based on scientific evidence, and focused on the promotion of human health^52^. The long-term mental and physical health of mothers, newborns and families must be considered when measuring outcomes and reshaping services, and mothers and their families must be recognized as stakeholders and be involved in all levels of decision-making. This is critical as countries are still dealing with, and will likely continue to deal with COVID-19 waves and new variants of the disease, which may disproportionately affect pregnant women. Other countries are already planning and implementing post-COVID-19 health system reforms.

This pandemic has been a magnifying lens for the existing harmful policies in maternity services but should also be an important impetus for a radical rethinking of the way maternity services are delivered in the future.

## Data Availability

The data supporting this research are available from the authors on reasonable request.
